# Effects of varying the standard deviation of the luminance on the appearance of food, flavour expectations, and taste/flavour perception

**DOI:** 10.1038/s41598-020-73189-8

**Published:** 2020-09-30

**Authors:** Junya Ueda, Charles Spence, Katsunori Okajima

**Affiliations:** 1 Yokohama National University, 79-7 Tokiwadai, Hodogaya, Yokohama, 240-8501 Japan; 2 University of Oxford, Anna Watts Building, Oxford, OX2 6GG UK

**Keywords:** Perception, Psychology

## Abstract

What we taste is affected by what we see, and that includes the colour, opacity, and shape of the food we consume. We report two experiments designed to investigate how the standard deviation (SD) of the luminance distribution of food images influences the perceived visual texture and the taste/flavour experience by using the latest Augmented Reality (AR) technology. We developed a novel AR system capable of modifying the luminance distribution of foods in real-time using dynamic image processing for simulating actual eating situations. Importantly, this form of dynamic image manipulation does not change the colour on the food (which has been studied extensively previously). Instead, the approach outlined here was used to change the SD of the luminance distribution of the food while keeping the chromaticity, the average luminance, and the skewness constant. We investigated the effects of changing the luminance SD distribution of Baumkuchen (a German baked cake) and tomato ketchup on visual perception, flavour expectations, and the ensuing taste experience. Participants looked at a piece of Baumkuchen (Experiment 1) or a spoonful of tomato ketchup (Experiment 2) having different luminance distributions and evaluated the taste on sampling the food. Manipulating the SD of the luminance distribution affected not only the expected taste/flavour of the food (e.g. expected moistness, wateriness and deliciousness), but also the actual taste properties on sampling the food itself. The novel food modification method and system outlined here can therefore potentially be used to control the taste/flavour of different foods crossmodally by means of modifying their appearance properties (specifically the SD of the luminance distribution while keeping other aspects of image statistics constant), and can do so in real time, without the need for food markers.

## Introduction

Generally-speaking, our choice of which foods to purchase and/or consume is determined by visual cues (i.e., by what we see). As Apicius, the Roman gourmand, once said “We eat first with our eyes”^1^. The visual appearance of food and drink is also very important as far as assessing its freshness is concerned^2–4^. Additionally, visual appearance cues can trigger the desire to eat^5–8^. In fact, well before a food is consumed, its appearance properties help to set expectations concerning the taste, flavour, and palatability of the food which ultimately affect its acceptance and thereafter its consumption^9^.

If the visual appearance of food can be modified at will, we may be able to control people’s appetite. Moreover, it may also be possible to improve the palatability of food, such as in the case of hospital foods^10,11^, or in those diet foods which intrinsically have little taste/flavour^12^. Relevant here, visual exposure to a novel food has been shown to be a particularly effective means of introducing new foods to children^12,13^. Neophobia, or the “fear of something new”, is an adaptive trait that typically peaks between the ages of two and five years of age. Crucially, neophobia has been shown to reduce the intake of fruits, vegetables, and meats in children^12–14^. Therefore, the appearance of novel food can potentially exert a significant influence over people’s food intake by helping to reduce food neophobia and hence possibly facilitating food acceptance.

Those children who have been exposed to pictures of novel foods, or actual foods, typically exhibit a greater willingness to try them^8,15,16^. Similarly, those children presented with a visually-familiar fruit before a novel one show a greater willingness to try the novel fruit as compared to those children who were only exposed to the novel fruit^17^. According to such results, if one were to modify the visual appearance of a food, it might be possible to eliminate, or at the very least to modify, children’s food dislikes based on the latter’s appearance.

On the other hand, many people consider taste to be the single most important sensory factor affecting food intake^18^. However, taste is not only affected by a food’s chemical composition (that directly influences the taste and smell, or retronasal olfaction, directly), but also by the information that is received via the other senses (including vision, audition, and touch, or oral-somatosensation). Typically, we see food first, then smell it orthonasally, and possibly also hear it being prepared (think only of the sizzle of the steak on the hotplate, or the sound of the coffee machine making that delicious cup of coffee)^19^. These various sensory cues can help to set our flavour expectations.

### Colour’s influence over taste and flavour expectations

Prior to ingestion, colour influences judgments of the acceptability of food by affecting expectations of palatability which can ultimately determine food choice and consumption^20–22^. For instance, a white wine will be described with more red wine-related adjectives when coloured red or rose, than when left uncoloured^23,24^. What is more, when distinct tastes are mixed in solution it has been reported that they are also more easily discernible when appropriately coloured than when no relevant colour cues are available. So, for instance, adding tasteless, odourless red colour to a clear solution significantly increased people’s perception of sweetness in the solution^25^. Similarly, adding green colouring significantly increased the detectability of sourness^26^. The association of red colour with sweetness and green with sourness is thought to be due to learned colour-flavour associations indicating that redness indicates ripeness or maturity of a fruit and green colour indicates immaturity lack of ripeness^20,26–29^. Adding colours to a solution can also aid in people’s flavour identification especially when atypical colour-taste combinations are presented. For instance, flavoured solutions mixed with an atypical colour were harder to identify than those mixed with a typical colour^29,30^. Therefore, prior experiences with flavours influence the accuracy of people’s flavour-colour identification responses^22^. Even when told to ignore the colour when trying to identify the flavour, people were more accurate in identifying the flavour when coloured rather than uncoloured or miscoloured solutions were presented, The latter result thus suggests that the use of colour as a visual cue to help identify a flavour may be an automatic process^31,32^. Typical and atypical combinations of colour and taste/flavour have also been shown to influence people’s judgments of the palatability of food^33,34^.

### Influence of luminance distribution on taste expectation/perception

The relationship between a food’s colour and the sense of taste has been extensively researched. At the same time, however, little is currently known about the effects of variations of the luminance distribution of food appearance on food/flavour perception. In addition, the luminance distribution of food changes during eating in real-time because the luminance values on the food depend on the positional relationship between the food and the ambient source of lighting. That said, it has been demonstrated that variations of the luminance distribution influence the appearance of freshness of cabbage in still images^35^. Therefore, it is necessary to clarify the effects of the luminance histogram of food to food appearance, flavour expectations, and taste perception by using dynamic images. It is important to stress the fact that our real-world food experiences tend to be dynamic.

The colour of food is used to predict the likely taste/flavour of food and drink^36^. The basic idea here is that we share several associations between sensory attributes, either physically present or else merely imagined, in different sensory modalities. So, for example, people map sour-tasting and carbonated foods and beverages onto sharper shapes, whereas they preferentially map creamy foods and still drinks onto more rounded shapes instead^36,37^.

Information technologies can be used in order to modify the appearance of food while keeping the food intact. As an example of applying AR technology in food, the AR Meta cookie adds an AR marker to a cookie and changes its appearance, for example, by transforming a plain cookie into what looks like a strawberry-flavoured cookie. The same technology has also been used to enlarge the apparent size of food^38–43^. However, it is important to stress that such systems rely on the presence of an AR marker on the food. This is simply not possible for many fresh foods, semi-liquid substances (such as ketchup) and drinks etc. We therefore developed a real-time disguise system for foods with dynamic image processing that crucially does not require the presence of an AR marker so as to be able to modify the appearance of fresh foods and drinks. The system can modify the visual texture, colour, and luminance properties of food quantitatively thus enabling us to create new crossmodal effects between vision and taste, and their impact on multisensory flavour perception.

There are many benefits of being able to manipulate food dynamically. Dynamic food is more realistic, more attention-capturing, and more likely to lead to the illusion that what is seen over the headset is what is being tasted^39^. In that sense, food and AR represent a potentially great combination. Although the widespread uptake of AR in daily life has so far proved difficult, it is convenient for the purpose of food development, experimentation, and innovation. The combination of food and AR can exceed the limitations of traditional food research.

## Apparatus and image processing

As mentioned above, visual factors such as colour (hue) and the visual texture of food are known to affect our perception of various foods. AR technology provides a great deal of flexibility in terms of dynamically changing the visual appearance characteristics of foods. Crucially, we have developed a marker-less AR system in order to control the luminance distribution of food in real-time. That is, no AR code is required, unlike in the case of the alternative Meta cookie-type systems that have been reported previously^40–43^. The system described here, can be applied to any food, including those that may be difficult to process physically, such as cakes and thick sauces. Additionally, this system can control the luminance distribution as well as the colour of food in a quantitative manner. Therefore, using such technology allows us to investigate the effects of visual texture on food perception from the point of view of the food’s luminance distribution, while keeping all other visual appearance factors constant.

**Figure 1:**
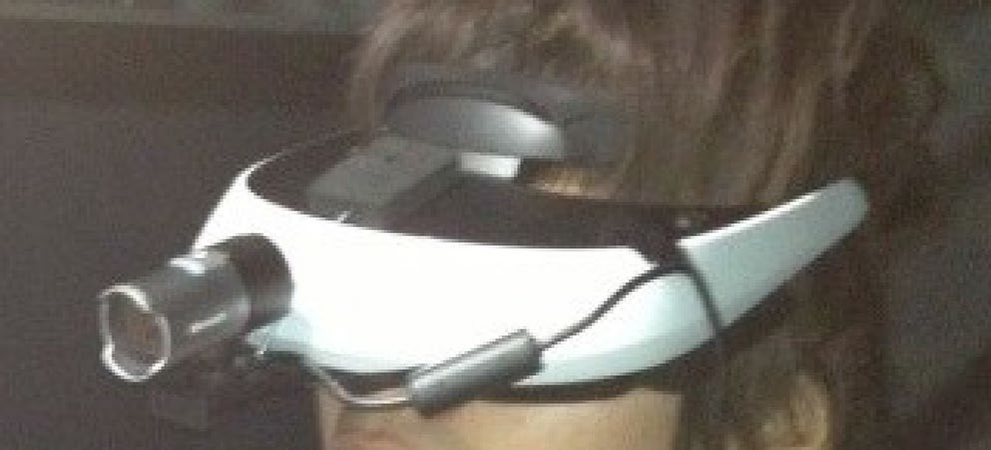
Apparatus (HMD and Camera) used in the food appearance modification experiments reported here.

Using this AR food modification system, we conducted a study in which the participants had to evaluate various different foods in order to study how changing the luminance distribution of the food affects not only its appearance but also taste and texture perception while keeping the average in luminance and chromaticity constant. The visual stimuli were displayed on a head-mounted display (SONY, HMZ-T2) with a camera (Microsoft, LifeCam Studio; see Fig. 1). This 1080p-HD camera has 8 million pixels. The image seen by the participants has a resolution of 1280 × 720 pixels. The image was displayed in full screen on the HMD. The luminance of the table’s surface was set to 270 cd/m^2^.

**Figure 2:**
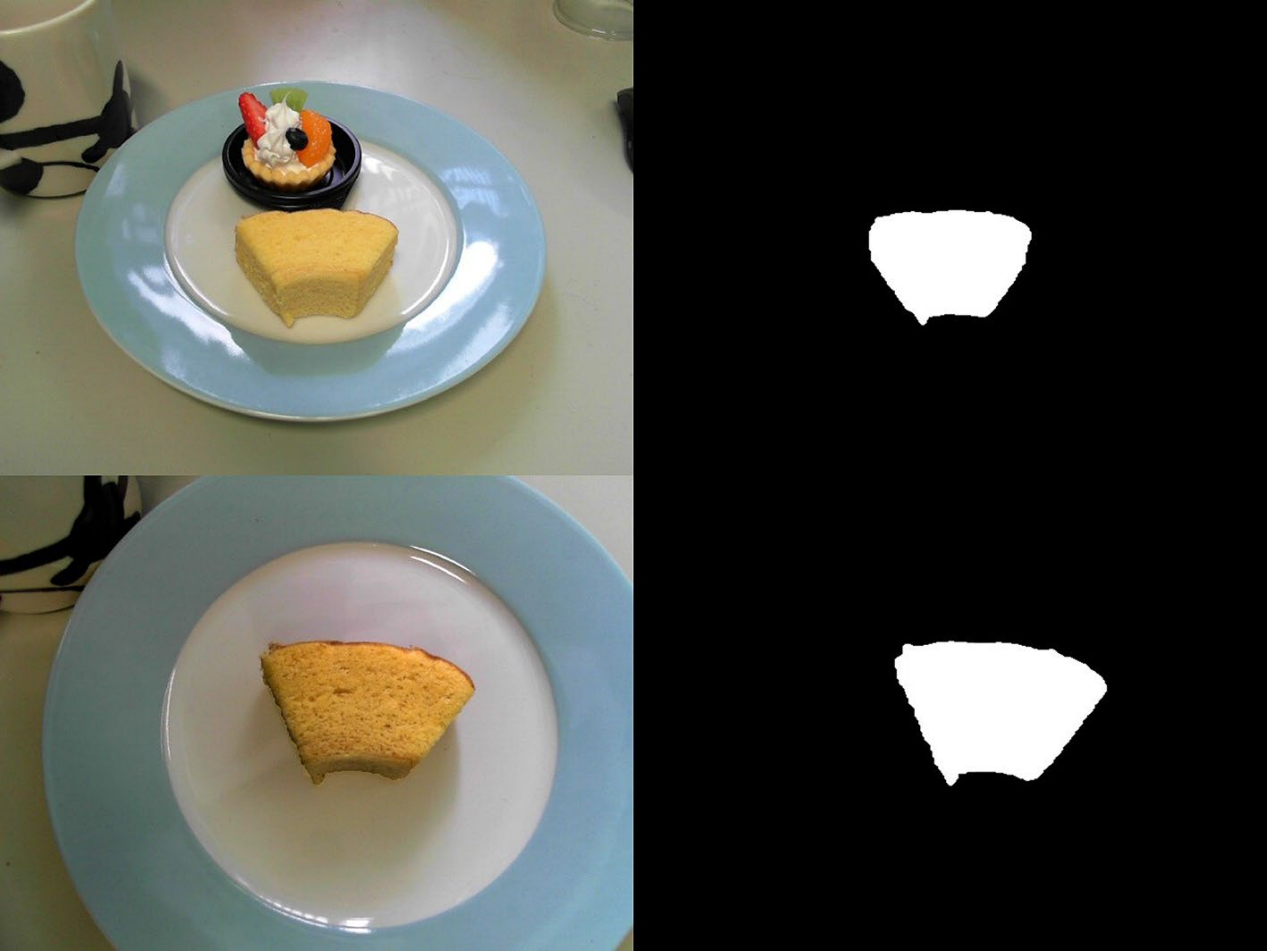
Original images (left), and results of extracted target food (right) in Experiment 1.

In order to quantify the crossmodal influences of the luminance distribution of the food image on people’s rating of the taste and oral texture of food, the appearance of a baked cake and (a spoonful of) tomato ketchup were changed by modifying their luminance SD. The resulting image was presented to participants in real-time by means of an HMD. We measured any crossmodal effects of modifying the SD of the luminance distribution on visual, taste and texture perception. This system used colour and size information of the target food to extract the food region from the input image provided by the camera^44^. Figure 2 illustrates the results of extracting the food region from a camera image.

After the food region had been extracted by the system, the luminance histogram of that region (Histogram-A) was calculated. In the histogram, the value (B) is calculated by magnifying the difference between a bin value (A) and the average value of the histogram (the magnification factor is *K*). The number of pixels (Number in bin-B) for the value (B) is 1/*K* times as large as the number in bin-A. If *K* exceeds one, the numbers of pixels for the values (B + 1), (B + 2),…, (B + *K*-1) is also set to the number in bin-B while keeping the sum of pixels and linearity of the histogram intact. For example, when *K* = 2, half of the number at bin-A was set to bins B and B + 1; when *K* = 3, one third of the number at bin -A was set to bins B, B + 1, and B + 2.

**Figure 3:**
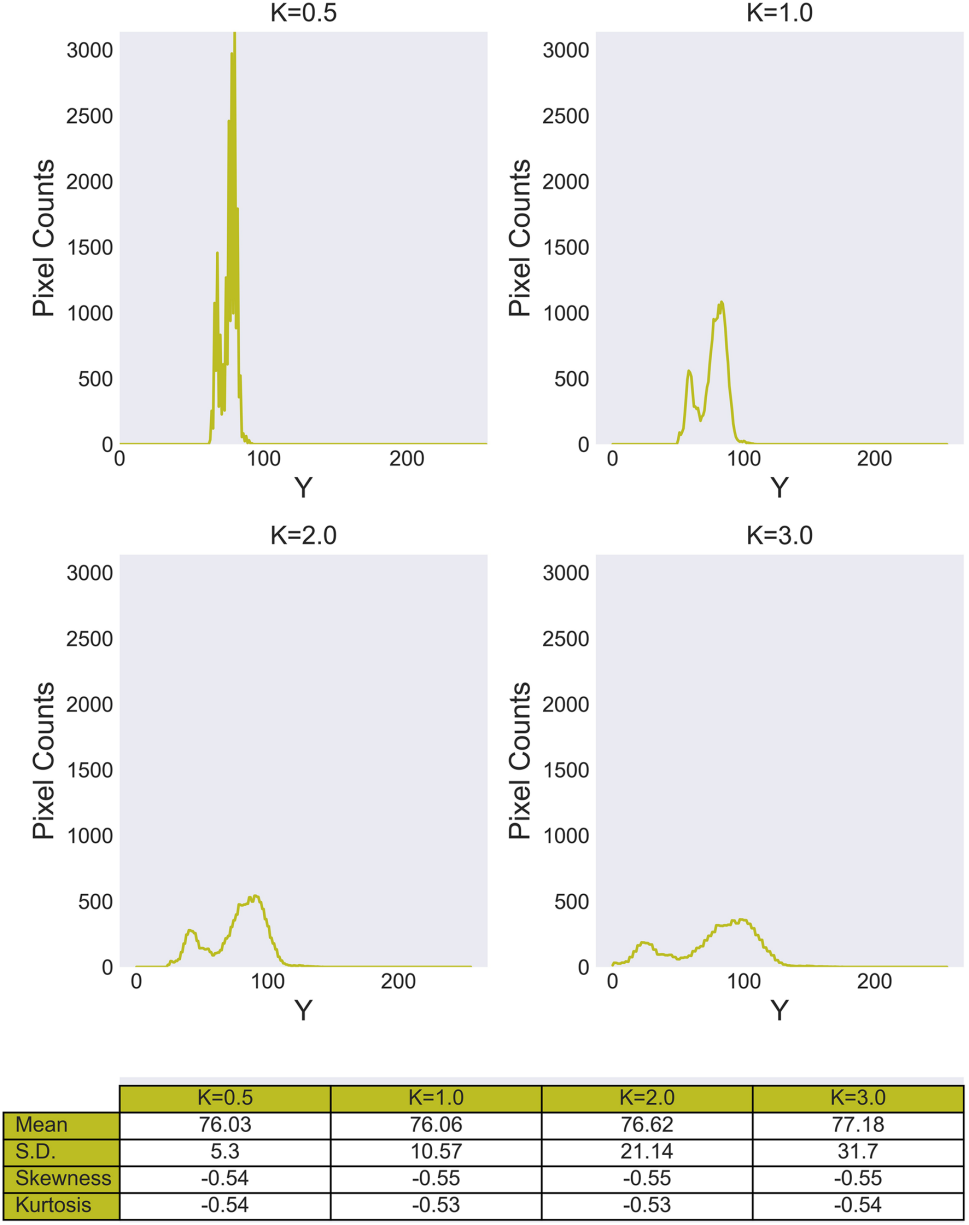
Examples of luminance histogram and associated statistical values. Y on the horizontal axis represents Y in XYZ tristimulus values.

These manipulations are executed repeatedly for all values of the original histogram (Histogram-A). Figure 3 highlights the example of the histogram changes for a frame and SD, kurtosis, and the skewness of the luminance histogram changed by modifying the luminance distribution. The system executed the above manipulation at every frame in real-time. We used different types of foods; one solid (Experiment 1) and the other semi-liquid (Experiment 2).

## Experiment 1: Baumkuchen

### Stimuli

**Figure 4:**
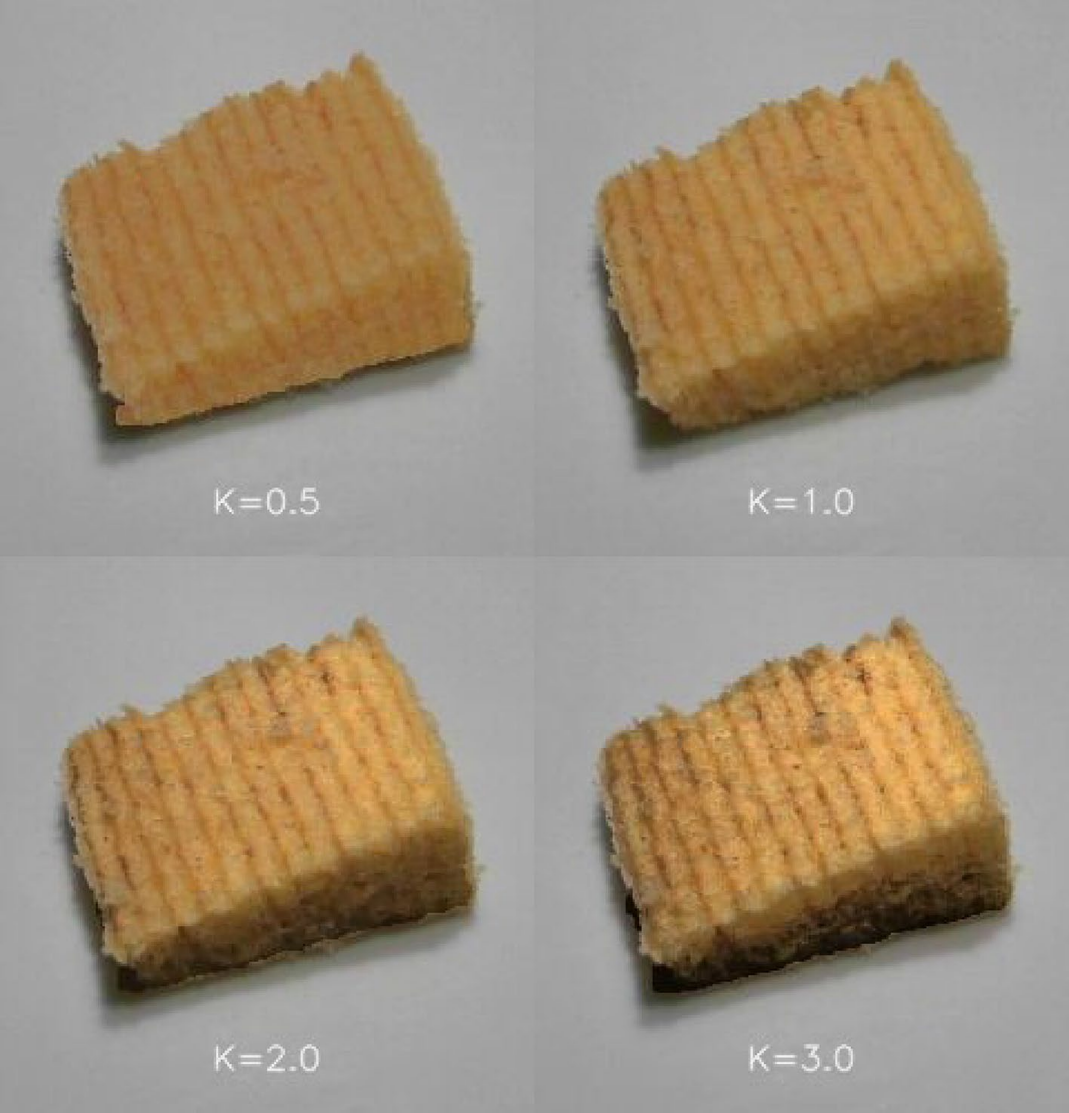
Modified luminance distribution of Baumkuchen A used in Experiment 1. The value *K* represents the magnification factor of the luminance histogram.

We investigated the effect of varying the visual texture on the oral-somatosensory perception of texture when sampling a food. We used Baumkuchen (a type of German baked cake that is popular in Japan) as one of the food stimuli. Baumkuchen constitutes an easy to evaluate (and recognize) texture for Japanese participants. The HMD was carefully calibrated with a colourimeter (Konica Minolta, CS-200). The luminance distribution of the original image was changed in three ways while, at the same time, the original chromaticity and the average luminance of the food image were kept constant. The luminance distribution area was expanded by 0.5, 2.0, and 3.0 times. Two kinds of Baumkuchen: Seven-Premium (A) and Nekkarich (B), which are widely sold at supermarkets and convenience stores across Japan were used as the experimental stimuli. In some of the trials, another brand of cake was used as a dummy stimulus, and the participants also evaluated it. We wanted to avoid the possibility of participants noticing that there were only two types of Baumkuchen and hence potentially polarizing their taste evaluations. We attempted to avoid this possibility by means of physically preparing another brand of Baumkuchen (as a dummy). It should, though, be noted that the third type of cake was not included in the data analysis. The reason for this exclusion was that we did not want our participants to consume too much food. Figure 4 shows an example of the modified luminance distributions in the image of the food stimuli (Baumkuchen). We made sure to ensure that the plates were always in the same colour because the plate colour has, independently, been shown to affect people’s sensory-discriminative and hedonic response to food^19,45–47^.

### Procedure

Each trial started with the participants rinsing their mouth out with water. Next, the participants viewed a stimulus that was modified in real time on the HMD. They were instructed to evaluate the expected taste and texture based solely on the basis of the food’s visual appearance. The participants then tasted the sample and evaluated both its taste and texture. The evaluation questions 1. Appearance prior to eating (i.e., measuring visual expectations) and 2. The evaluation question while actually eating the cake (measuring flavour perception) were always presented in the same order, matching the situation that occurs during everyday consumption. This study was designed to verify the effect of appearance when manipulated by means of AR. Nevertheless, one might wonder whether asking people explicitly about their expectations prior to tasting might somehow have emphasized this aspect (or draw attention explicitly to this element of the sensory impression) more than might otherwise normally be the case^9^. The participants were allowed to pick the food up freely using a fork while at the same time looking at it. We did not use the original visual texture condition (*K* = 1) because we were afraid that exposure to such a stimulus might prime the notion of a familiar Baumkuchen and thus possibly bias participants’ subsequent taste evaluations^48^. Each participant completed a total of 21 trials (3 luminance conditions × 3 repetitions × 2 kinds of cake + 3 dummy trials).

The participants evaluated the visual attributes of perceived moistness and deliciousness. The oral attributes that participants evaluated when they tasted the cake were perceived moistness, deliciousness, and sweetness. The participants evaluated the cake using an 11-point scale ranging from 0 to 10. The attributes and their scales are shown in Table 1.

**Table 1:** Estimated attributes and meanings of the scores for Baumkuchen.

	10 (max)	0 (min)
Moistness in appearance	Unbaked cheesecake	Dry sponge cake
Deliciousness in appearance	Looks delicious	Looks tasteless
Moistness in taste	Unbaked cheesecake	Dry sponge cake
Deliciousness in taste	Very delicious	Tastes bad
Sweetness in taste	Very sweet	Not at all sweet

Thirteen university students (ranging in age from 20 to 23 years) took part in the study. The participants were not aware of the aim of the study and that the appearance of food was modified. This research protocol was assessed prior to execution relative to the guidelines of Yokohama National University Committee on Life Science Research meeting all requirements for exemption from formal application for ethical review. This study was also performed in accordance with relevant guidelines and legislation. All participants consented to take part in the experiments in accordance with the guidelines.

### Results

**Figure 5:**
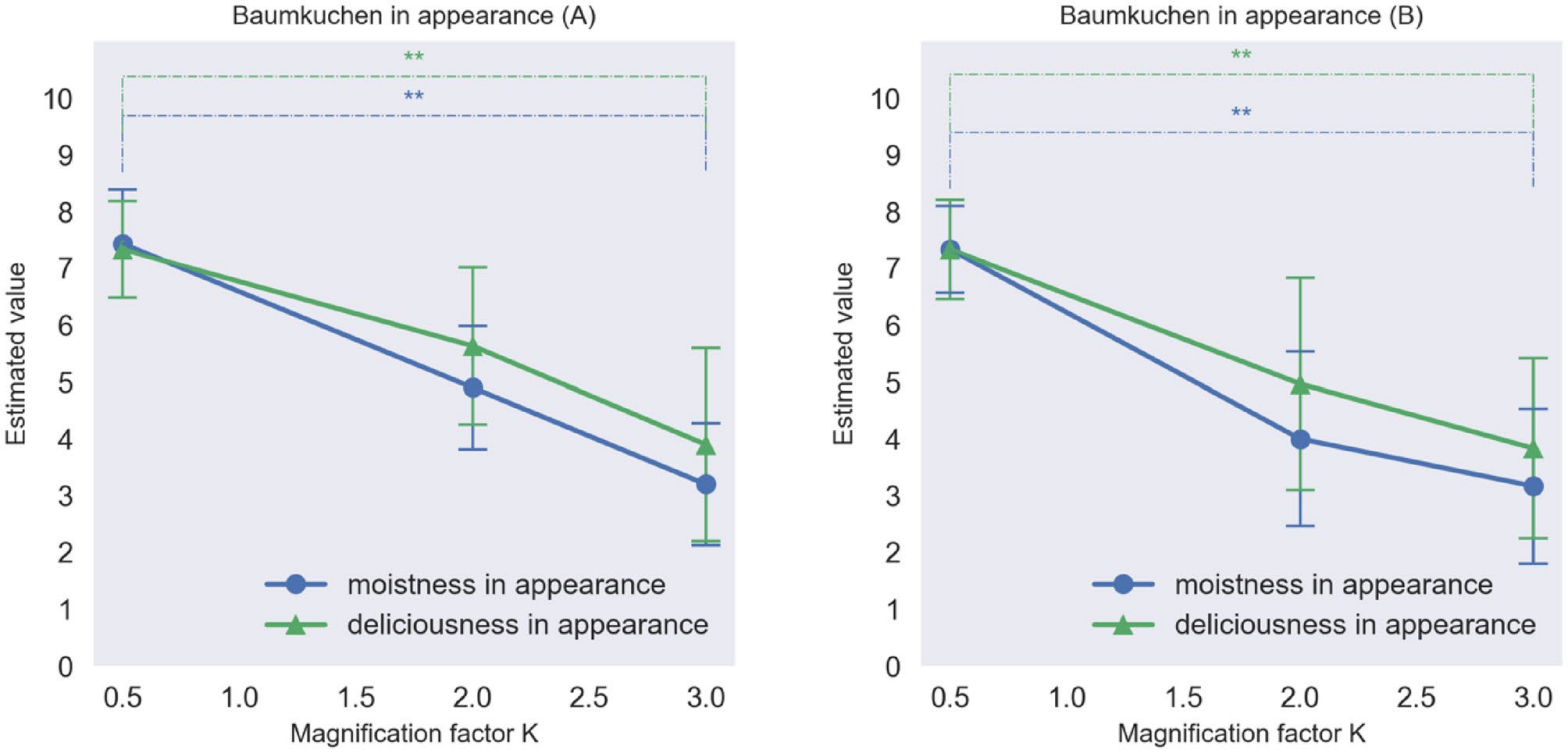
Moistness and deliciousness based on the appearance of the Baumkuchen A (left) and B (right). See Table1 for the meanings of the estimated value on y-axes (*p  < 0.05, **p < 0.01, Error bars represent the standard error).

**Figure 6:**
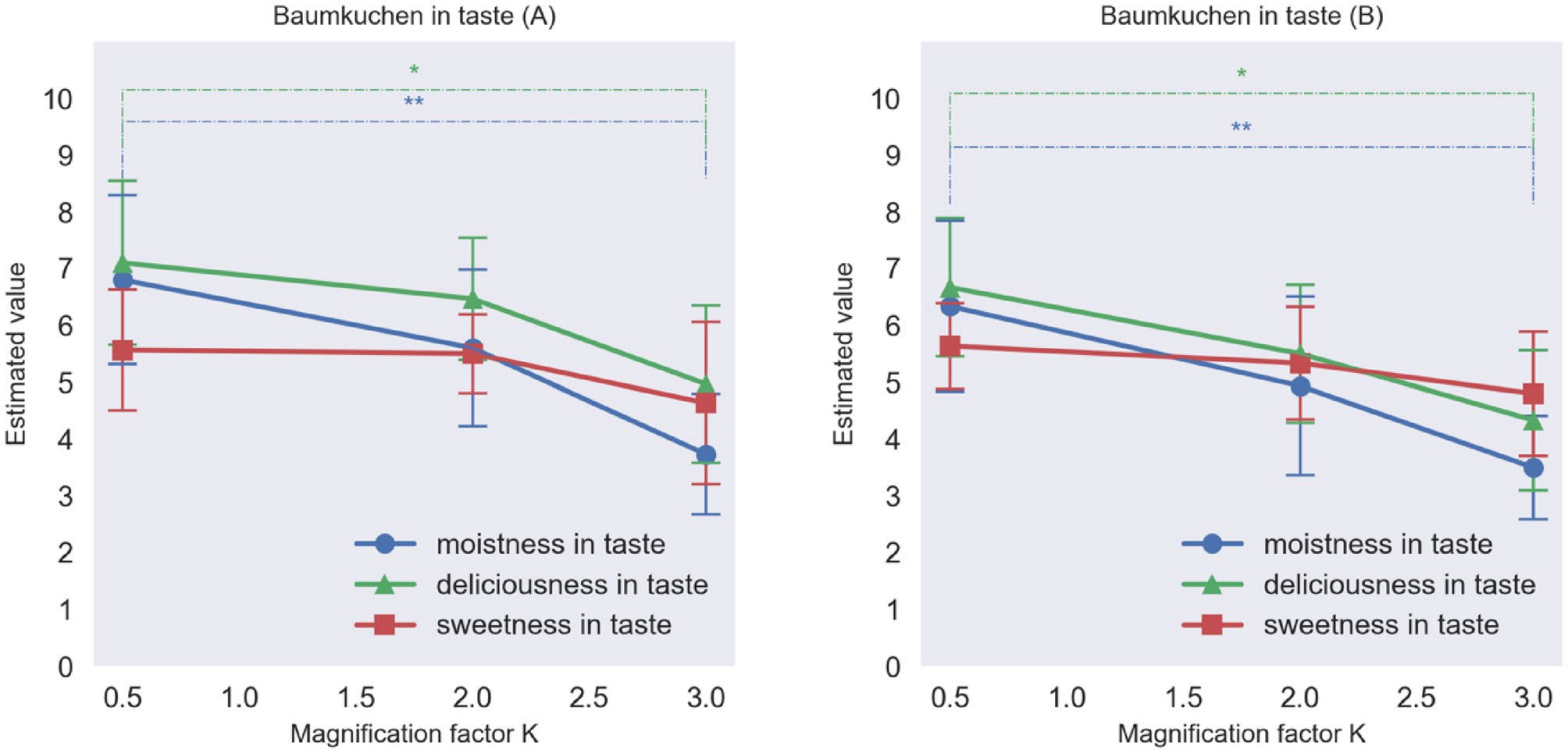
Moistness, deliciousness, and sweetness in taste of Baumkuchen A (left) and B (right) in Experiment 1. (*p  < 0.05, **p  < 0.01, Error bars represent the standard error).

Figure 5 shows the average moistness and deliciousness ratings of each sample of Baumkuchen at the magnification factors *K* = 0.5, 2.0, and 3.0. The results of an analysis of variance (ANOVA) revealed that for both kinds of Baumkuchen, moistness, and deliciousness in appearance changed significantly as a function of the modification of the luminance distributions of the food samples (p < 0.01) between the 0.5 and 3.0 conditions of the *K* factor. This result suggests that changing the SD of the luminance of the food can be used to modify the visual perception of the expected moistness and deliciousness of the food. Figure 6 shows the average moistness, deliciousness, and sweetness ratings for the Baumkuchen when sampled by the participants. The results indicate that for both kinds of cake, the moistness and deliciousness of the taste changed significantly as a function of the modification of the luminance distributions (p < 0.01) between the 0.5 and 3.0 conditions of the *K* factor. On the other hand, sweetness ratings did not change significantly. Despite the fact that the participants tasted one and the same Baumkuchen, it looked different, and the participants assigned different taste and texture ratings on sampling it. Figure 5 reveals that the moistness and deliciousness in appearance decreased with increasing the magnification factor *K* of the luminance histogram (Baumkuchen A: moistness F(2,27) = 21.43, p < 0.001, η_p_^2^ = 0.06; deliciousness, F(2,27) = 8.22, p = 0.002, η_p_2 = 0.04; Baumkuchen B: moistness F(2,27) = 15.53, p < 0.001, η_p_^2^ = 0.05; deliciousness F(2,27) = 7.22, p = 0.003, η_p_^2^ = 0.03). Figure 6 shows that moistness and deliciousness in terms of taste changed as the magnification factor K of the luminance histogram was increased (Baumkuchen A: moistness F (2,27) = 7.00, p = 0.004, η_p_^2^ = 0.03; deliciousness F(2,27) = 4.66, p = 0.018, η_p_^2^ = 0.02; Baumkuchen B: moistness F(2,27) = 5.55, p = 0.009, η_p_^2^ = 0.03; deliciousness F(2,27) = 4.66, p = 0.018, η_p_^2^ = 0.03). Sweetness in terms of taste did not depend on the magnification factor *K* (Baumkuchen A: F(2,27) = 1.14, p = 0.333, η_p_^2^ = 0.01; Baumkuchen B: F (2,27) = 1.00, p = 0.381, η_p_^2^ = 0.01). We performed the post hoc power analysis by using the G*Power software and the results revealed that the statistical power was 0.56 for the effect size of 0.4.

## Experiment 2: Tomato ketchup

### Stimuli

Tomato ketchup was used to measure the effect of visual texture (and hence flavour expectations) on oral-somatosensory texture perception when the ketchup was sampled by participants. Note that sauces represent a texture that is particularly difficult/challenging to add an AR marker to. Nevertheless, just as for Experiment 1, the luminance distribution of the original image was changed in three ways while keeping the original chromaticity and the average brightness of the food constant. The luminance distribution area was expanded by a factor of 0.5, 2.0, and 3.0. As the majority of participants are used to seeing ketchup in their everyday lives, we did not include the original texture (*K* = 1.0) in the experimental condition. We used two kinds of tomato ketchup (A) and (B) as the stimuli but another brand of tomato ketchup was used as a dummy in some of the trials. The participants evaluated the dummy, but these results were excluded from the analyses.

**Figure 7:**
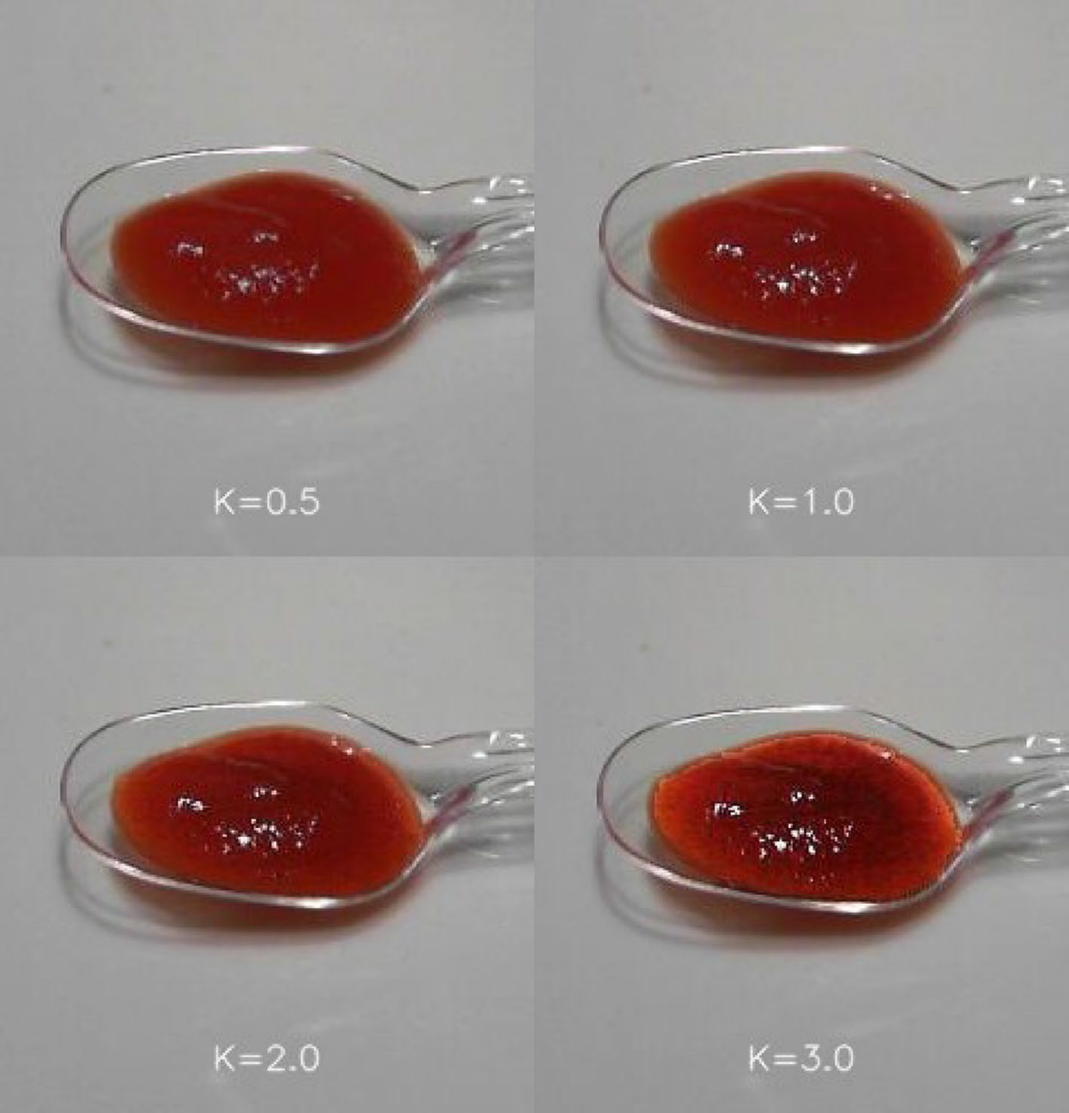
Example images of tomato ketchup with the original and modified luminance distributions used in Experiment 2.

Figure 7 shows examples of the modified luminance distribution of the stimuli. Notice that transparent plastic spoons were used to present the ketchup.

### Procedure

Thirteen university students (ranging in age from 20 to 23 years) took part in the study. The visual attributes that were evaluated by participants were watery and tomato flavour perception. The oral attributes in terms of taste/flavour were watery, tomato flavour, and acidity (see Table 2). The procedure was the same as Experiment 1.

**Table 2:** Estimated attributes and meanings of the scores for tomato ketchup in Experiment 2.

	10 (max)	0 (min)
Watery in appearance	Very watery	Not at all watery
Tomato flavour in appearance	Looks very strong	Looks very weak
Watery in taste	Very watery	Not at all watery
Tomato flavour in taste	Very strong	Very weak
Acidity in taste	Very sour	Not at all sour

### Results

**Figure 8:**
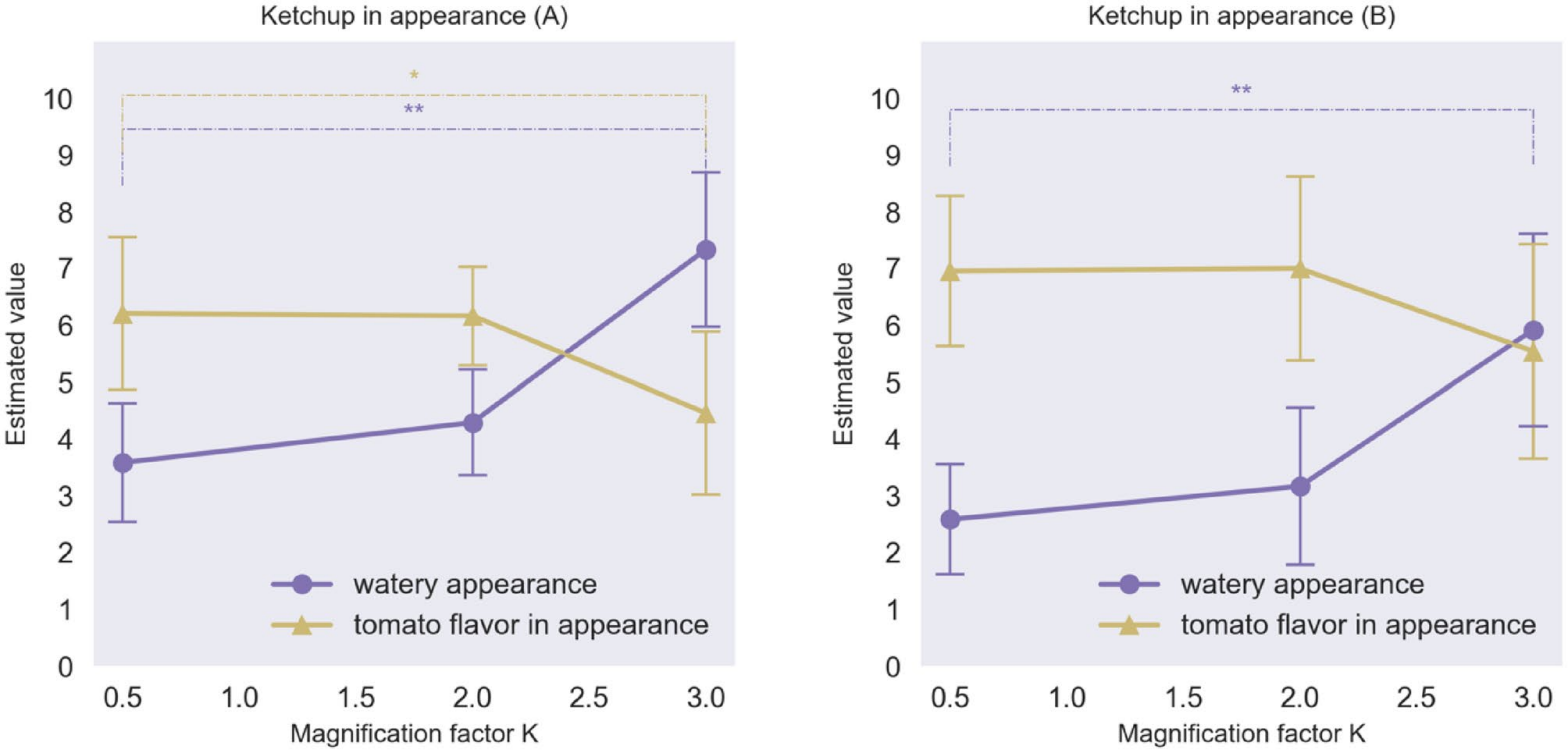
Watery appearance and expected tomato flavour based to the appearance of the Tomato Ketchup A (left) and B (right) in Experiment 2. (*p  < 0.05, **p  < 0.01, Error bars represent the standard error).

**Figure 9:**
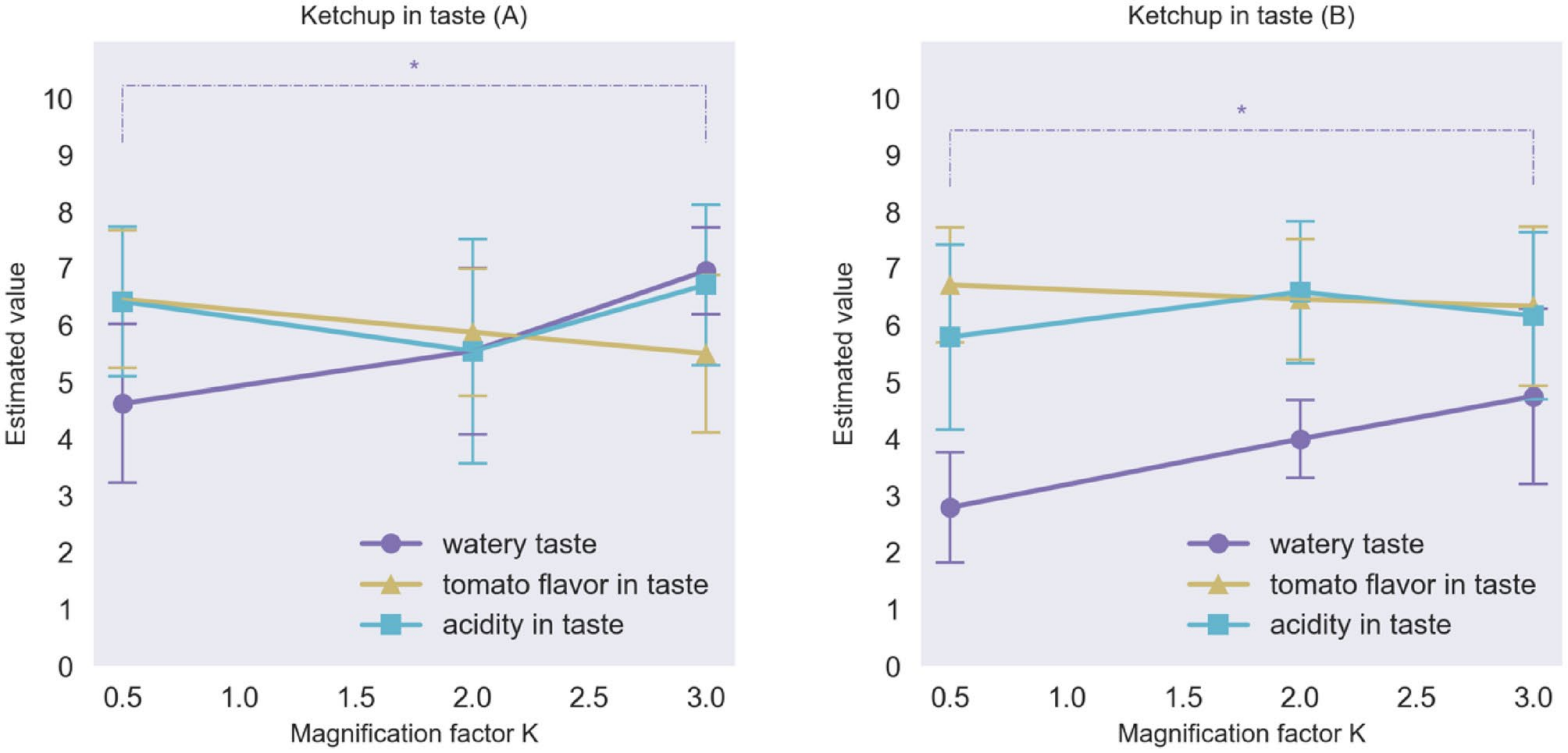
Watery taste, tomato flavour, and acidity in taste of Ketchup A (left) and B (right) in Experiment 2. (*p < 0.05, **p  < 0.01, Error bars represent the standard error).

Figure 8 shows the averages of watery judgments and tomato flavour for each kind of ketchup varying in terms of its appearance as a function of the magnification factor. The results of an ANOVA revealed that for both kinds of tomato ketchup, the watery appearance and the visual impression of tomato flavour changed as the luminance distributions were expanded (watery F(2,23) = 17.52, p < 0.0001, η_p_^2^ = 0.08; tomato flavour F(2, 23) = 3.62; p = 0.044, η_p_^2^ = 0.03) 2 or 3 times, thus suggesting that the SD of the luminance distribution also affects the visual perception of tomato ketchup between 0.5 and 3.0 conditions of the *K* factor. Figure 9 shows the average ratings of watery, tomato flavour, and acidity of the ketchup in terms of taste/flavour, indicating that the significant differences were only observed between the 0.5 and 3.0 conditions of the *K* factor. The results of tomato ketchup (A) indicate that watery in terms of taste changed as the luminance distributions were expanded (Ketchup A: watery F(2,23) = 4.98, p = 0.017, η_p_^2^ = 0.04; tomato flavour F(2,23) = 0.84, p = 0.446, η_p_^2^ = 0.01; acidity F(2,23) = 0.82, p = 0.456, η_p_^2^ = 0.01; Ketchup B: watery F(2,23) = 4.32, p = 0.027, η_p_^2^ = 0.04; tomato flavour F(2,23) = 0.15, p = 0.863, η_p_^2^ = 0.002, acidity F(2,23) = 0.41, p = 0.668, η_p_^2^ = 0.005) although it is worth noting that the magnitude of this change was smaller than that seen for the Baumkuchen. No significant differences in tomato flavour or acidity were observed. A post hoc power analysis using the G*Power software revealed that the statistical power was 0.56 for the effect size of 0.4.

## General discussion

The results of the two experiments reported in the present study demonstrate that the luminance SD of the food image affects the complex appearance properties of food, such as its expected moistness, wateriness and deliciousness. Crucially, only the luminance SD of foods was varied by applying the latest AR technology which requires no AR marker while keeping other statistics, such as the average and the skewness intact. Many previous studies have shown the effects of the colour (both hue and saturation) on food perception^49–51^. Importantly, however, in the present study, we made sure to keep the chromaticity of the both Baumkuchen and tomato ketchup constant. This allowed us to demonstrate for the first time that not only colour (hue), but also the luminance SD of the visible surface texture of the food can influence people’s perception of the oral-somatosensory texture as perceived when that food is sampled. Visual wetness can be predicted by image colour statistics^52^. However, we found that moistness and wateriness which are similar attributes to wetness can be modulated by altering only the luminance information just as for the perception of glossiness^53^. On the other hand, the effect of the luminance modulation on perceived sweetness was relatively modest. This suggests that the association between visual texture and sweetness is weak. A correlation between sweetness intensity and visual intensity has already been documented in a number of published studies^50–56^.

It should be noted that the significant differences across the 3 conditions were always found between the 0.5 and 3.0 conditions, and not between the 0.5 and 2.0 conditions, or between the 2.0 and 3.0 conditions. The moistness and deliciousness ratings shown in Fig. 6 and the watery taste data shown in Fig. 9 appear to vary monotonically as a function of the magnification factor *K*. However, it is important to stress that differences between 0.5 and 2.0 and between 2.0 and 3.0 conditions were too small to obtain statistically significant differences probably for individual differences among participants. In this study, 3.0 was used as the maximum value of the magnification factor *K*. Theoretically, however, it could be set to 4.0 or 5.0, and it is to be expected that significant difference between 1.0 (the original) and such a high value would be obtained.

The results reported here demonstrate that it is possible to change the taste/flavour of food crossmodally by modulating the SD of the luminance distribution associated with the image of the food. As yet, however, it is unclear whether such effects would also occur were the surround to be modulated in the same way. If the contrast of the global visual scene was to be modulated proportionally in the same way as reported in the present study, we speculate that it would appear just a blurred or sharp-edged image (world) depending on the magnification factor of the SD. One might also wonder whether mechanisms of perceptual constancy might start to kick-in. Therefore, we guess that such effects in this study do not occur when the surround was modulated in the same way too, and the local modulation of the luminance distribution of the food is critical for such effects in this study.

In the two experiments reported here, only the luminance distribution of the food images was converted. In the future, we may also be able to enhance the perceived sweetness of various food stimuli by means of dynamically modifying the virtual colour of the food, as has been shown previously by physically modifying the actual colour of the food itself^20,26,27^.

The findings and systems reported here will likely contribute to the study of the profound effect of visual appearance on the perception of food. Notice how the appearance of the food can be rapidly and repeatedly changed without having to modify the food physically/chemically. For example, it is possible to radically change the texture, to control the luminance distribution quantitatively, and/or to create a dynamic texture. These operations can be applied to many AR food applications. For example, improvement of likes and dislikes, dietary education of children, diet, improvement of taste of hospital food, and the like. It may also prove effective as a food design development tool for food companies.

## Conclusions

We report on the development of a real-time luminance modification system for foods with dynamic image processing and without the need for any AR marker to be placed on the food itself. The approach outlined here extracts the food region in real-time from an image using image features, colour, and edge detection. The modified images so created are both very stable and highly realistic. Using such a system, we were able to manipulate only the SD of the luminance distribution of the food while keeping the chromaticity (colour) and average luminance intensity (brightness) of the food constant. We found that the luminance SD of the food image affects the complex food appearance, such as moistness, wateriness and deliciousness expectation. As such, we were able to investigate for the first time the effects of modifying the luminance distribution on taste/flavour perception. The AR system described here can potentially be used to modify the taste and perceived texture of food simply by manipulating its appearance.

## Data Availability

The dataset generated and analysed during the current study are available from the corresponding author on reasonable request.
